# Contributions of prior health and vaccination to inequality in COVID-19 mortality by income: analysis of administrative microdata for the non-institutionalized Dutch population

**DOI:** 10.1093/ije/dyag188

**Published:** 2026-09-11

**Authors:** Elena Milkovska, Jawa Issa, Pieter van Baal, Bram Wouterse

**Affiliations:** Erasmus School of Health Policy and Management, Erasmus University Rotterdam, Rotterdam, The Netherlands; Erasmus School of Health Policy and Management, Erasmus University Rotterdam, Rotterdam, The Netherlands; Erasmus School of Health Policy and Management, Erasmus University Rotterdam, Rotterdam, The Netherlands; IQ Health, The Radboud University Medical Center, Nijmegen, The Netherlands

**Keywords:** COVID-19, healthcare disparities, health policy, public health, socio-economic factors

## Abstract

**Background:**

The COVID-19 pandemic caused a large health burden that disproportionally fell on people with a lower socio-economic status. Not much is known about the role of structural differences in pre-existing health and vaccination uptake in explaining these inequalities. We aim to disentangle these factors and quantify their contribution to income-related inequality in COVID-19 mortality.

**Methods:**

Using linked Dutch administrative microdata (*N* = 6 619 699), we model the risk of COVID-19 death based on pre-existing health and vaccination status for the entire population above the age of 50 years in 2021. Health is proxied by individual-level remaining life expectancy based on predictions by income using detailed information on demographics, specific outpatient medication(s), and hospital records. Building on this, we then quantify the extent to which differences in pre-existing health and vaccination status between lower and higher incomes explain income-related differences in COVID-19 deaths.

**Results:**

We find strong income-related differences in COVID-19 mortality. In 2021, individuals of the poorest income quintile were about three times more likely to die of COVID-19 than those in the richest quintile. Differences in pre-existing health are more important than differences in vaccination uptake in explaining this gap. For the poorest males/females, 67%/46% can be explained by differences in health and 14%/19% by vaccination status.

**Conclusion:**

Differences in pre-existing health by income are likely the most important determinant of income-related inequality in COVID-19 mortality. These findings signal that policies focused on reducing existing inequalities in health might lessen the burden of future health crises.

Key Messages

- We sought to assess the determinants of income inequalities in COVID-19 mortality in the Netherlands.
- Poor underlying health status played a greater role in explaining the higher mortality among people in lower-income groups than differences in vaccination uptake between low- and high-income groups.
- Our findings indicate that reducing socio-economic inequalities in health through policies that address structural and social determinants of health might lessen the burden of future health crises.

## Introduction

Socio-economic inequality in COVID-19 mortality has been well established in both low- and high-income countries [1]. The main identified drivers of socio-economic inequality in COVID-19 mortality are: pre-existing differences in health, differences in the availability/uptake of vaccination, unequal access to healthcare, and varying exposure to the virus [2, 3]. A better understanding of these drivers of the unequal health burden of COVID-19 is crucial for effective policymaking.

Despite the growing literature on the determinants of inequality in COVID-19 mortality, no study has quantified the contributions of different determinants by using individual-level data. Studies on the determinants of socio-economic inequality in COVID-19 mortality tend to focus on a single determinant and use aggregate-level data [4–10]. Studies have found that COVID-19 mortality was higher in areas with a high prevalence of chronic diseases—which are often deprived areas [4–6]. Furthermore, a higher share of essential workers and an overcrowded living situation have been identified as contributing causes for increased exposure to the virus for people in lower socio-economic classes [2, 7, 11, 12]. While studies have shown that vaccination uptake is higher in richer socio-economic groups [13–19], there is limited evidence on the effects of differences in vaccination on inequality in mortality. Previous studies using aggregate-level data from the USA and the UK investigated whether (i) vaccination has reduced inequalities, by comparing to a situation without vaccination, and (ii) more uptake of vaccination could further reduce inequalities. These suggested that vaccination dampened the link between socio-economic status and COVID-19 mortality [8, 9], while contradictory results from the USA suggested that vaccination aggravated inequality [10].

In this paper, we quantify the share of income-related inequality in COVID-19 mortality associated with differences in pre-existing health status and vaccination uptake. We use individual-level administrative data covering the entire Dutch population over the age of 50 years who are not residing in institutional care.

## Methods

### Data

We obtained administrative records from different sources for all residents of the Netherlands aged >50 years from 2011 to 2021 provided by Statistics Netherlands. We linked datasets at the individual level by using a pseudonymized personal identification number. Data from 2021 were used to identify COVID-19 deaths and vaccination status, while data from the pre-COVID-19 period (2011–18) were used to estimate a model to predict counterfactual life expectancy. Data from 2019 were used to classify the 2021 population into different income groups. We excluded residents of long-term care institutions because their health, COVID-19 exposure, and vaccination uptake were substantially different from those of the non-institutionalized population [20], resulting in a dataset of >6.6 million observations (Supplementary Figure II).

The National Institute for Public Health and the Environment provided COVID-19 vaccination records as reported in the national vaccination register (CIMS dataset), including vaccine name and date of vaccination. Individuals had to consent to be included in the register. This means that, in the data, we cannot distinguish between people who were not vaccinated and people who were vaccinated but did not give consent. For vaccinations performed by the municipal health services that performed 85% of all vaccinations, the overall share of vaccinated people giving consent was 93% of all vaccinations for the first vaccination and 95% for the booster. For the other 15% of vaccinations, provided by other parties such as general practitioners, there is no information about the share of individuals who gave consent.

We used the vaccination records to determine whether an individual had been fully vaccinated by 31 December 2021. People who were vaccinated but did not give permission were classified as not vaccinated. Full vaccination was defined as having received a full course of the chosen vaccination within the recommended timeline (see [20] and Supplementary Figure II). The vaccination campaign was first focused on individuals over the age of 60 years, individuals who had severe illnesses, as well as healthcare workers and caregivers [20].

We used the death register to determine the vital status of each individual and the date and main cause of each death, which was classified by using the 10^th^ revision of the WHO International Statistical Classification of Diseases (ICD-10 codes). We used codes U07.1 (confirmed) and U07.2 (suspected COVID-19) to identify COVID-19 deaths. We calculated the age as of 1 January 2021 and categorized individuals into single-year age groups plus a 100+ group. We obtained each individual’s annual household disposable income corrected for the household size and composition in 2019 from the tax register [21]. Within each sex and single-year age group, we categorized individuals into quintiles based on their income. Therefore, each income group had the same age composition and our estimates of income differences in COVID-19 mortality were not confounded by differences in (average) age across the income groups.

We used annual data on all prescriptions reimbursed by the Dutch mandatory health insurance system for 2011 and 2019 to identify prescribed outpatient medication, categorized by using the Anatomical Therapeutic Chemical (ATC) classification at the therapeutic subgroup level. We used the National Basic Register of Hospital Care to retrieve the hospital admissions record of each individual and extracted information on the main diagnosis, urgency, and length of stay (LOS) of each admission, as well as the number of admissions in 2011 and 2019. Data from the cause-of-death registry for the years 2012–19 were used to calculate survival for the 2011 population.

### Statistical analysis

We estimated the probability of COVID-19 death in the last 5 months of 2021, as 92% of all vaccinations had already taken place by 1 August (Supplementary Figure III). The likelihood of COVID-19 death was modelled as a function of health and vaccination status by using logit models stratified by sex and income quintile, allowing for differences in the association between health and vaccination status across the income (and sex) groups. The models were then used to decompose the differences in COVID-19 mortality between each of the first four income quintiles and the richest quintile into differences due to differences in pre-existing health, vaccination status, and other (unobserved) factors. These other factors, accounting for differences in COVID-19 mortality that cannot be explained by income differences in health and vaccination status, are likely related to differences in exposure and/or differences in healthcare access (which we could not observe in the data).

The decomposition can be seen as a counterfactual approach, in which the richest quintile was the reference group. We estimated by how much the difference in COVID-19 mortality between one of the poorer groups and the richest group would have reduced if the poorer group had had the same health or vaccination uptake as the richest and the difference in mortality that would still remain. We used the non-linear decomposition approach of Powers, Yoshioka, and Yun [22], which addresses path dependency (the fact that the share of the difference attributed to either health or vaccination depends on the order in which they are introduced in the decomposition)—an issue that arises with the decomposition of non-linear models (see Supplementary file).

Health was measured by using individual-level predictions of the remaining life expectancy of the entire population aged >51 years at the start of 2021. Following the procedure outlined in Milkovska *et al.* [23], prior healthcare use served as an input for a prediction Cox-ElasticNet survival model that included age (1-year indicators), outpatient medication (ATC code at the therapeutic level), and hospital admission records [urgency, LOS, and number of admissions for 18 International Shortlist For Hospital Morbidity Tabulation (ISHMT) chapters]. The survival models were stratified by sex and income decile, which implies that life-expectancy estimates are also influenced by unobserved income-related differences that correlate with mortality, such as lines of work and health behaviour.

Models were estimated by using 2011 healthcare-use data with a 7-year follow-up and validated on 2019 data [24, 25]. Using the models, we estimated the hazard ratio of each individual based on the 2019 values of the predictors, as this was the most recent year in which healthcare usage was unaffected by the pandemic, and combined it with the estimated baseline survival to predict the remaining life expectancy of the individual (see Supplementary file). This health proxy condenses all the information contained in the model regressors into one composite measure of health. Analyses were carried out in STATA 19.5. The Supplementary file contains more detailed results and several robustness analyses.

## Results

The characteristics of this population, stratified by sex and income quintile, are presented in Table 1. For each sex, the predicted remaining life expectancy increased when going from lower- to higher-income groups. Almost 0.9 out of 6.6 million in the older Dutch population had not been fully vaccinated by the end of 2021 (13.2%). There were no differences in vaccination between males and females, and the number of people vaccinated increased with income for both sexes. The probability of COVID-19 death decreased with increasing income.

**Table 1:** Characteristics of the Dutch population aged ≥51 years not resident in nursing homes by sex and (age-standardized) income quintile groups. Remaining life expectancy was predicted from health indicators based on previous healthcare utilization, income, and sex.

		Population (*n*)	Mean age (years)	Mean health-predicted remaining life expectancy (years)	Vaccinated by end of 2021 [*n* (%^a^)]	COVID-19 deaths in 2021 [*n* (%^a^)]
Male						
	Q1 (poorest)	640 466	65.12	14.62	501 309 (78.3)	1040 (0.16)
	Q2	640 400	65.12	15.95	551 625 (86.1)	789 (0.12)
	Q3	640 371	65.12	16.71	567 246 (88.6)	661 (0.1)
	Q4	640 361	65.12	17.31	578 470 (90.3)	540 (0.08)
	Q5 (richest)	640 351	65.12	18.01	587 453 (91.7)	375 (0.06)
	All males	3 201 949	65.12	16.55	2 786 103 (87)	3405 (0.11)
Female						
	Q1 (poorest)	683 646	66.11	15.38	530 222 (77.6)	682 (0.1)
	Q2	683 556	66.11	15.82	585 531 (85.7)	499 (0.07)
	Q3	683 529	66.11	16.22	603 529 (88.3)	421 (0.06)
	Q4	683 518	66.11	16.59	615 572 (90.1)	308 (0.05)
	Q5 (richest)	683 501	66.11	17.43	625 484 (91.5)	253 (0.04)
	All females	3 417 750	66.11	16.29	2 960 338 (86.6)	2163 (0.06)
Overall		6 619 699	65.63	16.40	5 746 441 (86.8)	5568 (0.08)

a Between brackets: within row percentage.

**Figure 1:**
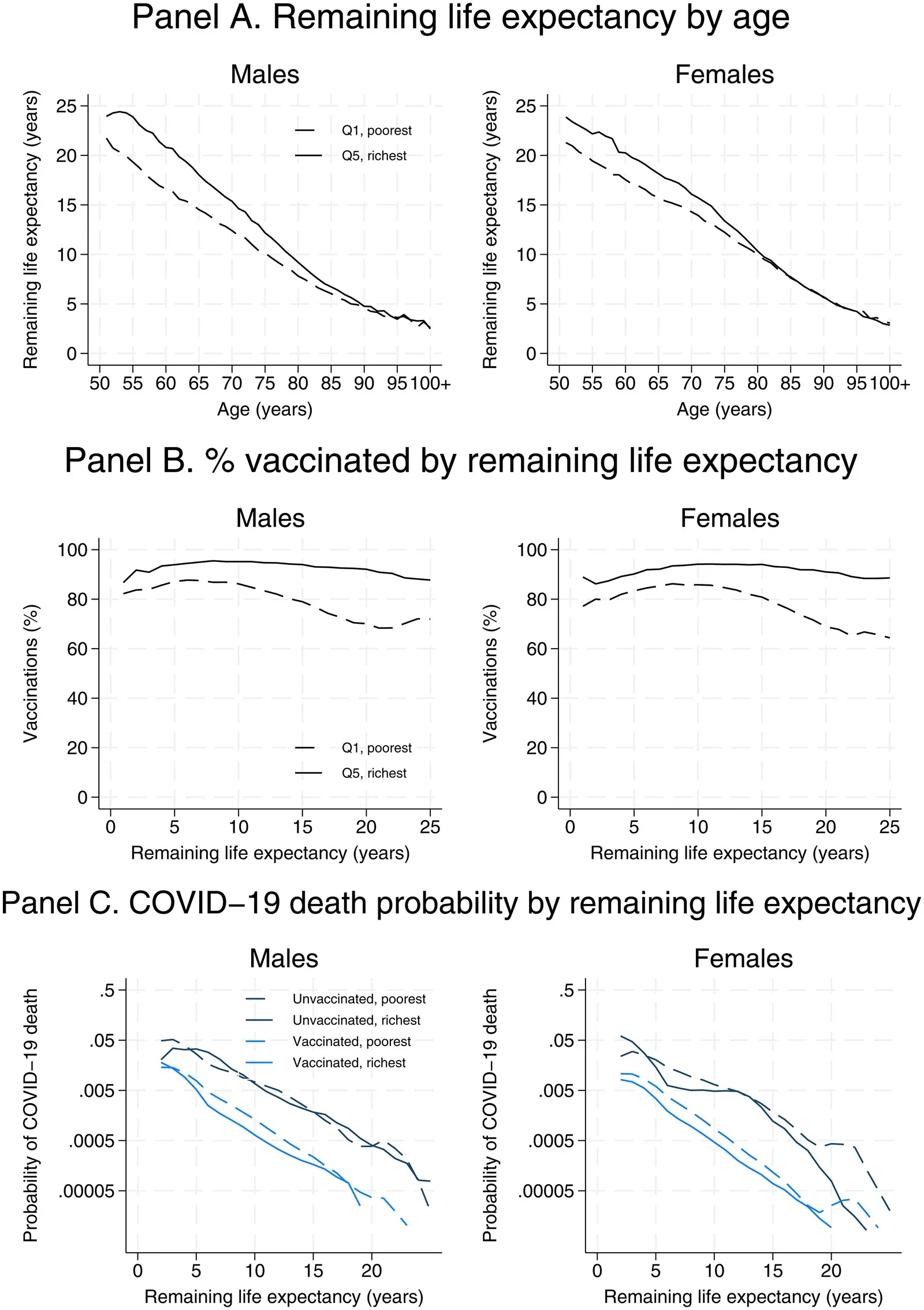
(A) Average remaining life expectancy by sex, age, and income quintile. (B) Vaccination distribution across the poorest and richest income quintiles by remaining life expectancy. (C) Average (smoothed) probability of COVID-19 death (presented on a logarithmic scale) for unvaccinated and vaccinated individuals in the poorest and richest quintiles by remaining life expectancy. Smoothing across LE was necessary to conceal small groups and retain anonymity per Statistics Netherlands policy. LE, remaining life expectancy.

The elements that potentially drive the income differences in COVID-19 mortality are shown in Fig. 1. As shown in Fig. 1A, the poorest individuals have lower remaining life expectancy than their richest counterparts across all ages, although the gap narrows at older ages.

As presented in Fig. 1B, across all levels of remaining life expectancy, there is an income gradient in vaccination uptake: conditional upon having similar pre-existing health, individuals with a lower income were less likely to be vaccinated. Interestingly, the income difference in vaccination status is larger in those with better health, with the vaccination status declining for high levels of life expectancy for lower incomes while staying stable for the richest. The lower vaccination uptake among those with zero to three remaining years of life expectancy could be an indication that very frail individuals were not encouraged to be vaccinated [26].

As shown in Fig. 1C, at all levels of remaining life expectancy, unvaccinated people were more likely to die from COVID-19. Conditional upon health and vaccination status, there was little difference in the probability of dying from COVID-19 between the poorest and richest groups.

The estimates of the stratified logistic models used for decomposition are presented in Table 2. We find that dying from COVID-19 is strongly associated with not being vaccinated. Considering the low probability of COVID-19 death in the population, the reported odds ratio approximates the risk ratio, showing that being vaccinated is associated with 79%–86% protection. Additionally, having one more year of predicted remaining life expectancy corresponds to a lower COVID-19 mortality risk on average by just over 25%.

**Table 2:** Logistic models of COVID-19 death in 2021 stratified by income quintile and sex.

	Poorest quintile	second quintile	third quintile	fourth quintile	Richest quintile
	Odds ratio (95% CI)	Odds ratio (95% CI)	Odds ratio (95% CI)	Odds ratio (95% CI)	Odds ratio (95% CI)
Male					
Vaccinated (0, 1)	0.208 (0.184–0.235)	0.184 (0.159–0.213)	0.162 (0.138–0.19)	0.16 (0.133–0.192)	0.138 (0.111–0.172)
Remaining life expectancy (years)	0.74 (0.729–0.75)	0.738 (0.726–0.749)	0.737 (0.724–0.75)	0.729 (0.716–0.743)	0.72 (0.703–0.736)
Constant	0.139 (0.119–0.162)	0.158 (0.132–0.189)	0.169 (0.14–0.204)	0.178 (0.144–0.221)	0.173 (0.135–0.223)
Pseudo *r*-squared	0.155	0.177	0.192	0.204	0.219
Female					
Vaccinated (0, 1)	0.172 (0.148–0.2)	0.174 (0.146–0.208)	0.178 (0.146–0.217)	0.158 (0.126–0.199)	0.14 (0.108–0.181)
Remaining life expectancy (years)	0.747 (0.735–0.759)	0.734 (0.72–0.748)	0.724 (0.708–0.739)	0.708 (0.69–0.727)	0.712 (0.691–0.733)
Constant	0.108 (0.09–0.13)	0.112 (0.091–0.139)	0.117 (0.093–0.147)	0.122 (0.094–0.158)	0.113 (0.085–0.149)
Pseudo *r*-squared	0.160	0.174	0.187	0.215	0.224

**Figure 2:**
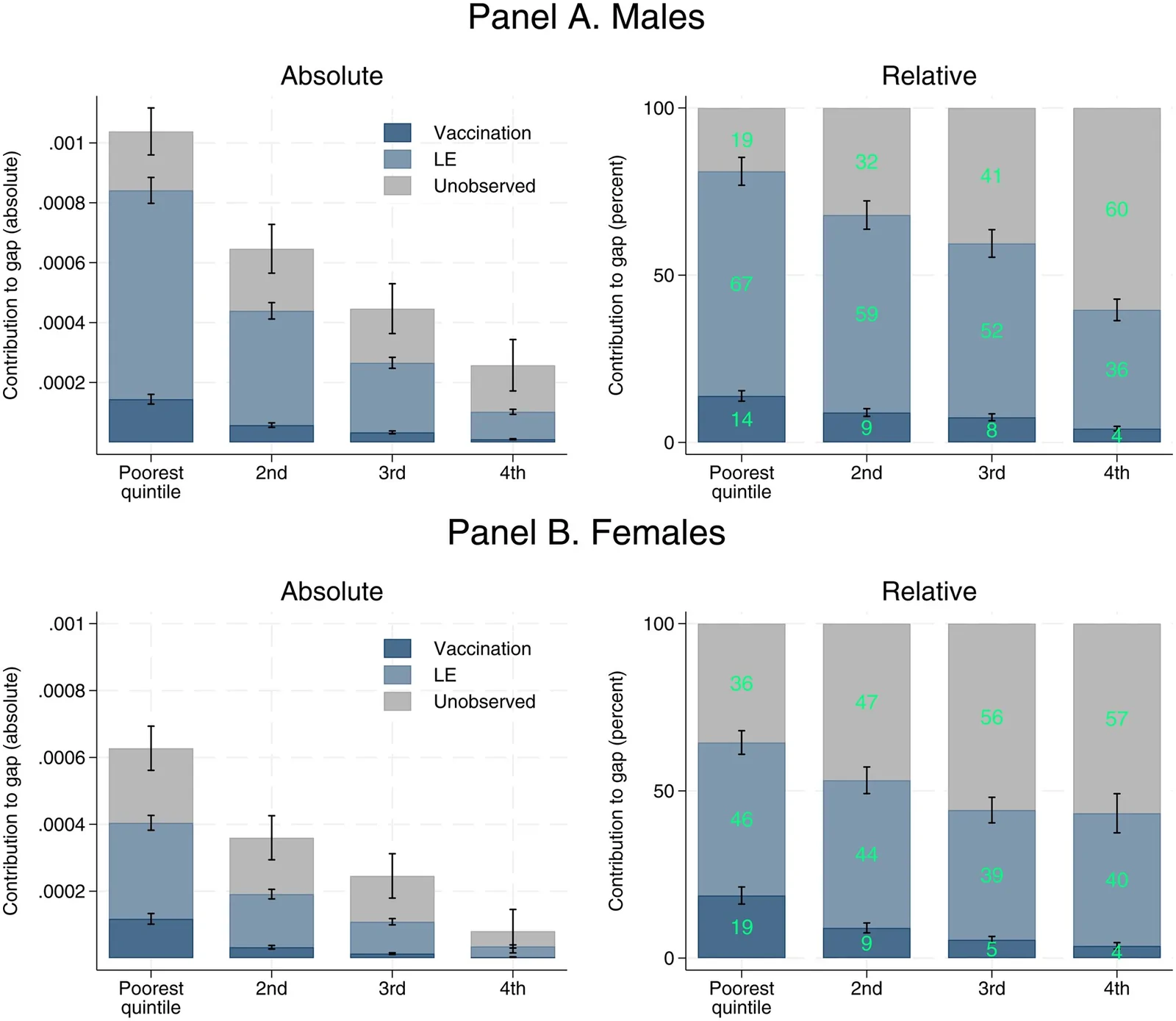
Non-linear decomposition of the income gap in COVID-19 mortality probability between the first four income quintiles and the richest quintile stratified by sex. Results are shown on the left in absolute terms and on the right in relative terms (percentage; error bar for the remainder group omitted). See Supplementary Table II for detailed results and confidence intervals. LE, remaining life expectancy.

Finally, the results for the decomposition of the inequality gap in the COVID-19 probability of death between the first four quintiles and the richest (fifth) quintile separately for males and females are shown in Fig. 2. While the absolute differences are smaller for females than for males, the overall pattern is similar: COVID-19 mortality, and thus the difference with the richest groups, decreases with income. The most important factor driving this inequality is the differences in prior health. For the lowest-income group, this explains 0.07 percentage points for males and 0.029 percentage points for females, while differences in vaccination status explain 0.014 percentage points and 0.012 percentage points for males and females, respectively. For higher-income groups, which have a more similar health and vaccination status to those of the richest groups, the relative importance of the unexplained factors in explaining the (small) gap in COVID-19 mortality does become more important (see Supplementary Table I). These results appear be robust both to a more flexible parameterization of the effect of remaining life expectancy and in an analysis restricted to ages of ≥65 years (see Supplementary Table II and Figure VI).

## Discussion

Using detailed microdata for the entire Dutch population aged ≥51 years, we found that the income-related inequalities in COVID-19 mortality in the last 5 months of 2021 were strongly related to differences in pre-existing health and, to a lesser extent, to differences in vaccination uptake. The role of other determinants, such as exposure and unequal access to healthcare, seemed limited. Pre-existing differences in health (measured by using predicted remaining life expectancy by income) explained two-thirds of the gap between the poorest and richest quintiles for males and for almost half of the females. Differences in vaccination explained under a fifth of the gap between the poorest and richest quintiles for both males and females. As access to and quality of care are highly equitable in the Netherlands (insurance is mandatory, out-of-pockets are low [27, 28]), we hypothesize that any remaining unexplained differences were likely due to differences in exposure to the virus.

The use of individual-level data on health and vaccination represents a notable methodological strength compared with previous studies that used aggregate-level data [8–10]. However, our study confirms the findings of these studies. Using a disability index at the county level, Chen *et al.* [10] found that differences in both health status and vaccination uptake by socio-economic status contributed to differences in the COVID-19 case fatality in the USA [10]. Even though their outcome measure is different and access to healthcare is likely to have played an important role in the USA, their findings underscore the relevance of pre-existing differences in health in shaping the health consequences of the pandemic. Sá [8] assessed the importance of various factors on COVID-19 mortality, including aggregate-level pre-existing health proxied by the average number of outpatient appointments. They found that vaccination uptake weakened the link between COVID-19 mortality and income and pre-existing health status. Their implied counterfactual was a situation in which nobody was vaccinated. Our findings also clearly suggest that, without vaccination, there would have been even stronger differences in COVID-19 mortality by income given the strong differences in pre-existing health status by income. Finally, Goto *et al.* [9] found that increasing the county-level vaccination rate could dampen the relationship between the county-level poverty rate and the COVID-19 death rate—which aligned with our findings. In this paper, we argue that existing income-related inequalities in health have contributed the most to inequalities in COVID-19 deaths. These pre-existing inequalities in health are caused by different mechanisms that run in both directions (from income to health and vice versa) and should be addressed by health as well as social policies [29, 30].

We recognize some limitations to our study, the most prominent of which stem from possible measurement errors in life expectancy and vaccination uptake. While we made strides in incorporating health status, it is important to recognize that our measure of health is a prediction of a composite metric of health based on individuals’ healthcare utilization that probably imperfectly proxies the underlying health status. Moreover, even with the use of individual-level data to produce this composite measure, some heterogeneity in health across individuals, even within income groups, may have been masked, as our predictors cannot fully capture all elements that affect the severity of health problems. For example, a decrease in life expectancy by 1 year due to cancer has different effects than 1 year due to kidney failure. Nevertheless, as survival models were stratified by income, we have included some unobserved differences by income both in health that is not reflected in healthcare use (such as obesity and smoking) and outside of health (such as behaviour effects or lines of work). Results based on a robustness analysis with lung cancer mortality as an outcome (see Supplementary Table II and Supplementary Figure VI) suggest that vaccination status might be associated with underlying health characteristics beyond COVID-19 vaccination itself, which may indicate that some residual confounding has influenced our findings. Additionally, while our survival models predicting life expectancy are not perfectly calibrated, the amount of mis-calibration is similar across the income groups (Supplementary Table III) and should therefore had a limited impact on our decomposition findings. Lastly, because, in the second half of 2021, the excess mortality attributed to COVID-19 at a population level exceeded the registered COVID-19 mortality [31], we might have underestimated the impact of COVID-19 on mortality and the potential inequalities therein. Data collection regarding vaccination was consensual and individuals could have chosen not to share their information. While Statistics Netherlands estimates that 93% of individuals accepted sharing information on initial vaccination and 95% on booster vaccination [32], vaccination status may still have been misclassified because we considered those who withheld consent as unvaccinated. This would have led to an underestimation of the effectiveness of vaccination, but the extent and direction to which this would bias the estimate of the role of vaccination in explaining income differences in COVID-19 mortality depend on whether the under-reporting was concentrated among the rich or the poor. However, the fact that our estimates of vaccine effectiveness are somewhat lower for the lower-income groups could be an indication that these people were less willing to share information about their vaccination status. If this is the case, then the effectiveness of vaccination is underestimated for the lowest-income group and the difference in the prevalence of vaccination between the rich and the poor is overestimated. On balance, this could imply an underestimation of the contribution of vaccination to income-related inequalities in COVID-19 mortality if the first effect dominates the latter. However, in the absence of any evidence on this correlation, we think it is more likely that our unexplained part in the decomposition would be lowered as a result of the misclassification of vaccination status.

Another limitation concerns unmeasured immunity [33]. If immunity is correlated with our health measure (e.g. individuals in poorer health might have been more/less likely to be infected or because immune response differs by health), then its effect would be attributed to the health component in the decomposition, otherwise it represents a measurement error. If immunity varies by income, then it would contribute to the unexplained component. Finally, if it is associated with vaccination status, then the direction of this bias would depend on whether immune individuals were more or less likely to get vaccinated. Although the determinants of COVID-19 vaccine uptake have been studied in the Netherlands [34, 35], the role of prior infection has not been examined and the net impact of this potential bias remains unclear.

Another limitation is that we excluded individuals in long-term care from the study, which accounted for a third of the COVID-19 deaths in 2021. Considering that lower-income individuals utilize more long-term care and have, on average, poorer health [36–38], including them would probably lead to a higher relative importance of health status when explaining income-related inequalities in COVID-19 mortality.

In conclusion, our findings highlight the importance of pre-existing health differences by socio-economic status, as they have crucially shaped the outcomes of the COVID-19 pandemic. These findings carry implications for policymakers, suggesting a need for addressing the structural inequalities in health.

## Ethics approval

There was no patient or public involvement, so no ethical approval was required.

## Supplementary Material

dyag188_Supplementary_Data

## Data Availability

The data underlying this article cannot be shared publicly due to privacy reasons. The data can be made available for researchers by the Centraal Bureau voor de Statistiek (Statistics Netherlands) through a remote access environment. To access the data, researchers must submit a research proposal to Statistics Netherlands (microdata@cbs.nl) and pay a fee. Upon request, the authors are willing to provide the estimated parameters from fitting distributions to the age-, sex-, and income-specific life expectancies.

## References

[1] McGowan VJ , Bambra C. COVID-19 mortality and deprivation: pandemic, syndemic, and endemic health inequalities. Lancet Public Health 2022;7:e966–e975. 10.1016/S2468-2667(22)00223-7 PMC9629845 36334610

[2] Patel JA , Nielsen FBH, Badiani AA et al Poverty, inequality and COVID-19: the forgotten vulnerable. Public Health 2020;183:110–1. 10.1016/j.puhe.2020.05.006 PMC7221360 32502699

[3] Abrams EM , Szefler SJ. COVID-19 and the impact of social determinants of health. Lancet Respir Med 2020;8:659–61. 10.1016/S2213-2600(20)30234-4 PMC7234789 32437646

[4] Semenzato L , Botton J, Drouin J et al Chronic diseases, health conditions and risk of COVID-19-related hospitalization and in-hospital mortality during the first wave of the epidemic in France: a cohort study of 66 million people. Lancet Reg Health Eur 2021;8:100158. 10.1016/j.lanepe.2021.100158 PMC8282330 34308411

[5] Noor FM , Islam MM. Prevalence and associated risk factors of mortality among COVID-19 patients: a meta-analysis. J Community Health 2020;45:1270–82. 10.1007/s10900-020-00920-x PMC7486583 32918645

[6] Islam N , Lacey B, Shabnam S et al Social inequality and the syndemic of chronic disease and COVID-19: county-level analysis in the USA. J Epidemiol Community Health 2021;75:496–500. 10.1136/jech-2020-215626 33402397

[7] Brandily P , Brébion C, Briole S et al A poorly understood disease? The impact of COVID-19 on the income gradient in mortality over the course of the pandemic. Eur Econ Rev 2021;140:103923. 10.1016/j.euroecorev.2021.103923 PMC8492390 34629487

[8] Sá F. Do vaccinations reduce inequality in Covid-19 mortality? evidence from England. Soc Sci Med 2022;305:115072. 10.1016/j.socscimed.2022.115072 PMC9132381 35640447

[9] Goto R , Kawachi I, Kondo N et al Contribution of vaccinations to reducing socioeconomic disparities in COVID-19 deaths across U.S. counties. Ann Epidemiol 2023;86:65–71.e3. 10.1016/j.annepidem.2023.07.003 37454832

[10] Chen Y , Zhang L, Li T et al Amplified effect of social vulnerability on health inequality regarding COVID-19 mortality in the USA: the mediating role of vaccination allocation. BMC Public Health 2022;22:2131. 10.1186/s12889-022-14592-w PMC9675971 36402963

[11] Harlem G. Descriptive analysis of social determinant factors in urban communities affected by COVID-19. J Public Health (Oxf) 2020;42:466–9. 10.1093/pubmed/fdaa078 PMC7313894 32530033

[12] Kontokosta CE , Hong B, Bonczak BJ. Socio-spatial inequality and the effects of density on COVID-19 transmission in US cities. Nat Cities 2024;1:83–93. 10.1038/s44284-023-00008-2.

[13] Dolby T , Finning K, Baker A et al Monitoring sociodemographic inequality in COVID-19 vaccination uptake in England: a national linked data study. J Epidemiol Community Health 2022;76:646–52. 10.1136/jech-2021-218415 35470259

[14] Spetz M , Lundberg L, Nwaru C et al An intersectional analysis of sociodemographic disparities in Covid-19 vaccination: a nationwide register-based study in Sweden. Vaccine 2022;40:6640–8. 10.1016/j.vaccine.2022.09.065 PMC9515344 36210254

[15] Nafilyan V , Dolby T, Razieh C et al Sociodemographic inequality in COVID-19 vaccination coverage among elderly adults in England: a national linked data study. BMJ Open 2021;11:e053402. 10.1136/bmjopen-2021-053402 PMC8313303 34301672

[16] Cavillot L , Loenhout JAF, van Devleesschauwer B et al Sociodemographic and socioeconomic disparities in COVID-19 vaccine uptake in Belgium: a nationwide record linkage study. J Epidemiol Community Health 2024;78:176–83. 10.1136/jech-2023-220751 PMC11045363 38148149

[17] Perry M , Akbari A, Cottrell S et al Inequalities in coverage of COVID-19 vaccination: A population register based cross-sectional study in Wales, UK. Vaccine 2021;39:6256–61. 10.1016/j.vaccine.2021.09.019 PMC8423991 34544601

[18] Attonito J , Van Arsdale W, Fishman K, Darya M, Jacomino M, Luck G. Sociodemographic disparities in access to COVID-19 vaccines upon initial rollout in Florida. Health Aff (Millwood) 2021;40:1883–91. 10.1377/hlthaff.2021.01055 34871075

[19] Williams AM , Clayton HB, Singleton JA. Racial and ethnic disparities in COVID-19 vaccination coverage: the contribution of socioeconomic and demographic factors. Am J Prev Med 2022;62:473–82. 10.1016/j.amepre.2021.10.008 PMC8598940 34872772

[20] Pluijmaekers A , Melker H de. *The National Immunisation Programme in the Netherlands. Surveillance and Developments in 2021–2022*. Published online 17 Nov. 2022. 10.21945/RIVM-2022-0042

[21] CBS. Equivalentiefactoren 1995–2000. CBS. 2004. https://www.cbs.nl/nl-nl/achtergrond/2004/25/equivalentiefactoren-1995-2000 (6 March 2026, date last accessed).

[22] Powers DA , Yoshioka H, Yun MS. Mvdcmp: multivariate decomposition for nonlinear response models. Stata J 2011;11:556–76. 10.1177/1536867X1201100404.

[23] Milkovska E , Wouterse B, Issa J et al Quantifying the health burden of COVID-19 using individual estimates of years of life lost based on population-wide administrative level data. Epidemiology 2025;36:520–30. 10.1097/EDE.0000000000001854 PMC12118614 40202801

[24] Deryugina T , Heutel G, Miller NH et al The mortality and medical costs of air pollution: evidence from changes in wind direction. Am Econ Rev 2019;109:4178–219. 10.1257/aer.20180279 PMC7080189 32189719

[25] Hanlon P , Chadwick F, Shah A et al COVID-19 - exploring the implications of long-term condition type and extent of multimorbidity on years of life lost: a modelling study [version 3; peer review: 3 approved]. Wellcome Open Res 2021;5:75. 10.12688/wellcomeopenres.15849.3 PMC7927210 33709037

[26] Yan Z , Yang M, Lai CL et al COVID-19 vaccinations: a comprehensive review of their safety and efficacy in special populations. Vaccines 2021;9:1097. 10.3390/vaccines9101097 PMC8539110 34696205

[27] OECD, Policies EO on HS and Netherlands: Country Health Profile 2017. *State of Health in the EU* State of Health in the EU, 2017;2017. 10.1787/9789264283503-en.

[28] Loef B , Meulman I, Herber GCM et al Socioeconomic differences in healthcare expenditure and utilization in The Netherlands. BMC Health Serv Res 2021;21:643. 10.1186/s12913-021-06694-9 PMC8254290 34217287

[29] Marmot M. The influence of income on health: views of an epidemiologist. Health Aff (Millwood) 2002;21:31–46. 10.1377/hlthaff.21.2.31 11900185

[30] Deaton A. Policy implications of the gradient of health and wealth. Health Aff (Millwood) 2002;21:13–30. 10.1377/hlthaff.21.2.13 11900153

[31] CBS RIVM. Sterfte en oversterfte in 2020 en 2021. CBS. 2022. https://www.cbs.nl/nl-nl/longread/rapportages/2022/sterfte-en-oversterfte-in-2020-en-2021 (13 July 2026, date last accessed).

[32] CIMS. COVID-vaccinatie Informatie- en Monitoringssysteem. CBS. n.d. https://www.cbs.nl/nl-nl/onze-diensten/maatwerk-en-microdata/microdata-zelf-onderzoek-doen/microdatabestanden/cims-covid-vaccinatie-informatie-en-monitoringssysteem (6 Mar. 2026, date last accessed).

[33] Oja AE , Saris A, Ghandour CA et al Divergent SARS-CoV-2-specific T- and B-cell responses in severe but not mild COVID-19 patients. Eur J Immunol 2020;50:1998–2012. 10.1002/EJI.202048908; PAGE: STRING: ARTICLE/CHAPTER 33073359

[34] Pijpers J , Roon A, van Roekel C, van et al Determinants of COVID-19 vaccine uptake in The Netherlands: a nationwide registry-based study. Vaccines (Basel) 2023;11:1409. 10.3390/VACCINES11091409/S1 PMC10537724 37766087

[35] Roekel C V , Labuschagne L, Pijpers J et al Factors associated with COVID-19 autumn 2022 booster uptake in the Netherlands among older adults aged ≥ 60 years and younger adults with chronic conditions. Vaccine 2024;42:146–55. 10.1016/J.VACCINE.2023.12.027. 38101955

[36] Abbing J , Suanet B, Groenou MB, Van. Socio-economic inequality in long-term care: a comparison of three time periods in the Netherlands. Ageing Soc 2023;43:643–63. 10.1017/S0144686X21000647.

[37] Wouterse B , Ram F, Baal P, van. Quality-adjusted life-years lost due to COVID-19 mortality: methods and application for The Netherlands. Value Health 2022;25:731–5. 10.1016/j.jval.2021.12.008 PMC8810280 35500946

[38] Issa J, , Wouterse B, , Milkovska E et al Quantifying income inequality in years of life lost to COVID-19: a predictionmodel approach using Dutch administrative data. Int Jepidemiol 2024;53. 10.1093/ije/dyad159 PMC10859130 38081182

