# Supplementary material for: Contributions of prior health and vaccination to inequality in COVID-19 mortality by income: analysis of administrative microdata for the non-institutionalized Dutch population
Source: Int J Epidemiol. 2026 Sep 11;55(5):dyag188. doi: 10.1093/ije/dyag188 (PMC13568478; doi:10.1093/ije/dyag188)
Supplement: Supplementary file 1 [file dyag188_supplementary_data.zip › ije-2026-03-0396-File005.pdf]

SUPPLEMENTAL MATERIAL

Contributions of Prior Health and Vaccination to Inequality in COVID-19 Mortality by Income: Analysis of Administrative Microdata for the non-institutionalized Dutch Population

Elena Milkovska (0000-0003-1143-4915), Jawa Issa, Pieter van Baal, Bram Wouterse

Contents

Population and exposure 2
More descriptives 5
More results and scenario analyses 7
Non-linear decomposition 13
Remaining life expectancy (LE) 14
Calibration 14
References 16

Supplementary Figure I Flowchart describing how vaccination status was determined 2
Supplementary Figure II Sample selection. 3
Supplementary Figure III Density of full COVID-19 vaccination by income quintile over 2021. 4
Supplementary Figure IV Average remaining life expectancy by sex, age and income quintile. 5
Supplementary Figure V Average probability of COVID-19 death (logarithmic scale) 6
Supplementary Figure VI Scenario analyses – LE squared, ages 65+, lung cancer 11
Supplementary Figure VII Scenario analyses – no LTFU and a lower bound analysis. 12

Supplementary Table I Non-Linear decomposition of the income gap in COVID-19 mortality 7
Supplementary Table II Regression results from various scenario analyses 10
Supplementary Table III Survival model performance measures on the validation dataset 15

Population and exposure

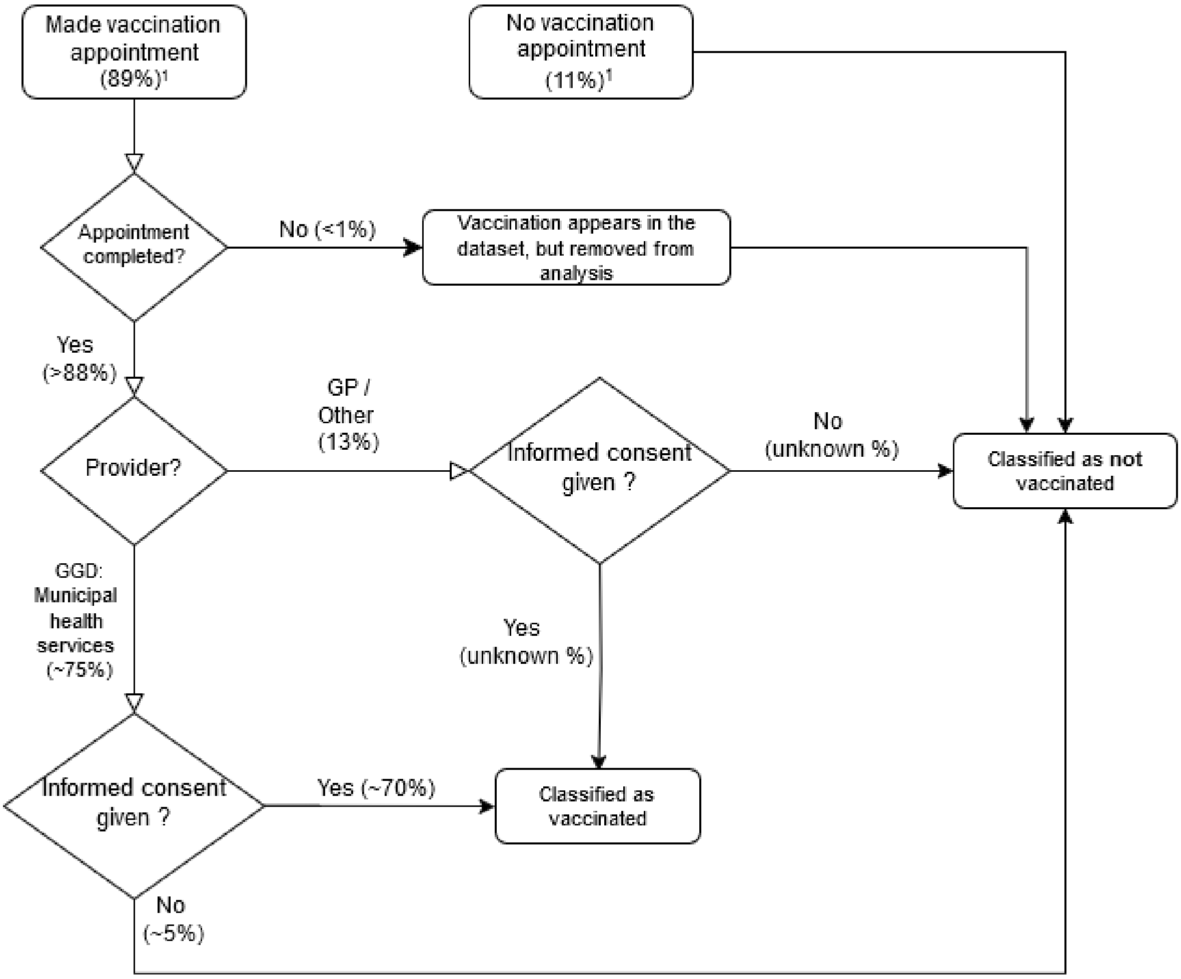

Supplementary Figure I Flowchart describing how vaccination status was determined using CIMS data.

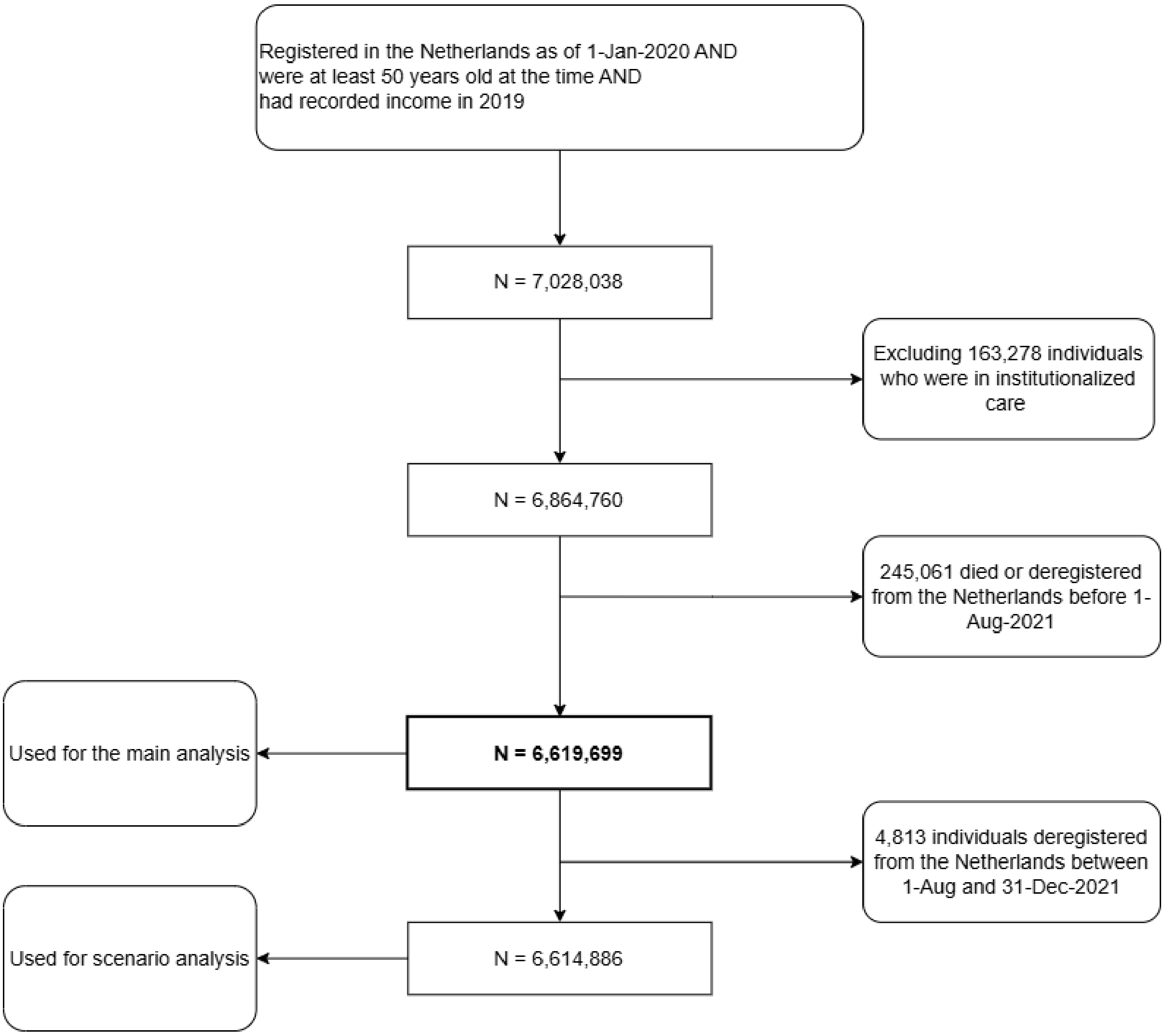

Supplementary Figure II Sample selection.

Our sample consists of people who were 1) registered in the Netherlands on 1-Jan-2020; 2) were at least 50 years old at the time; 3) had a recorded income in 2019 4) were not in institutionalized care 5) survived and remained registered in the NL until 1st Aug 2021. Several factors play into our subsetting choice:

- Life expectancy constraints - our model that predicts life expectancy can only do so for ages 50+ and does so by using 1-year lagged income and healthcare use data. Given this, we chose to observe registered people on 1-Jan-2020 so that we can use their pre-pandemic covariates when predicting life expectancy.

- Vaccination constraints – our main analysis looks at deaths occurring only in the last five months of 2021 - 91.9% of the vaccinations had already taken place by 1-Aug-2021 (see Figure III). Supplementary Figure VII shows a scenario analysis in where we consider the "lower bound" case where all individuals who got vaccinated after 1-Aug-2021 are assumed to be unvaccinated, and where we removed 4813 individuals who moved away between 1-Aug and 31-Dec-2021.

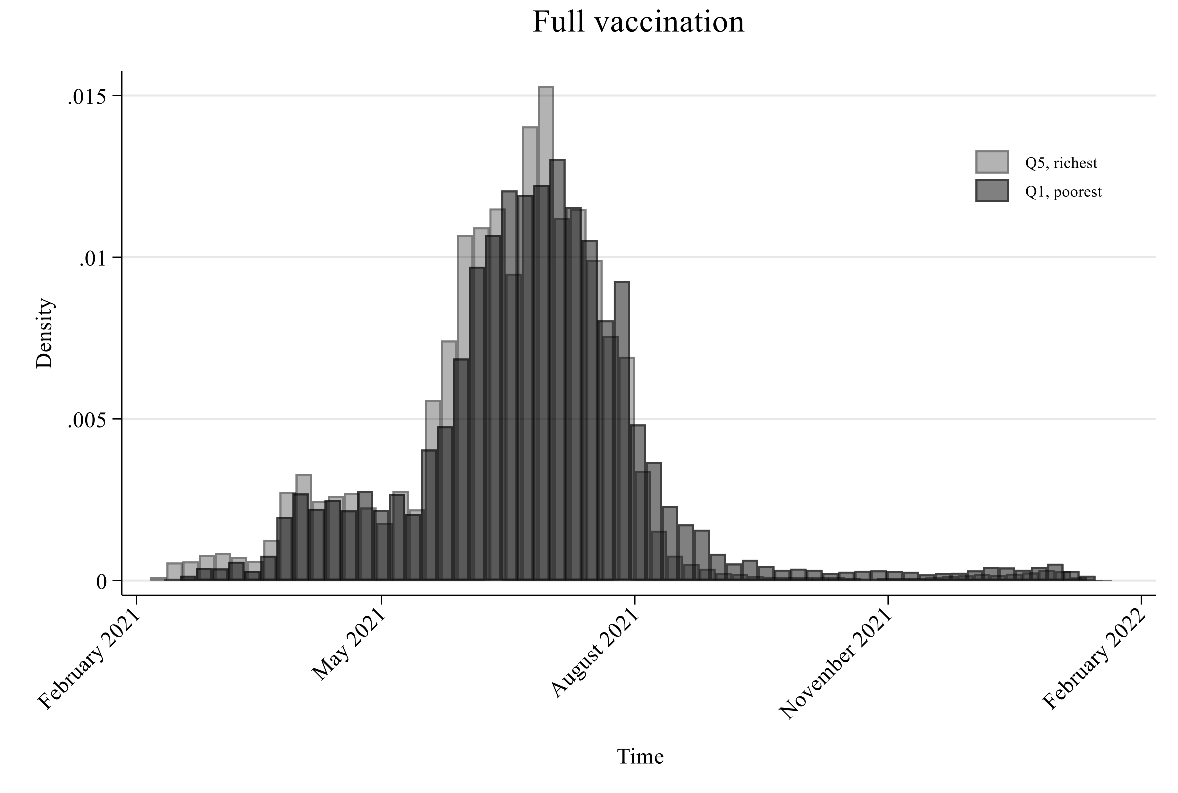

Supplementary Figure III Density of full COVID-19 vaccination by income quintile over 2021.

More descriptives

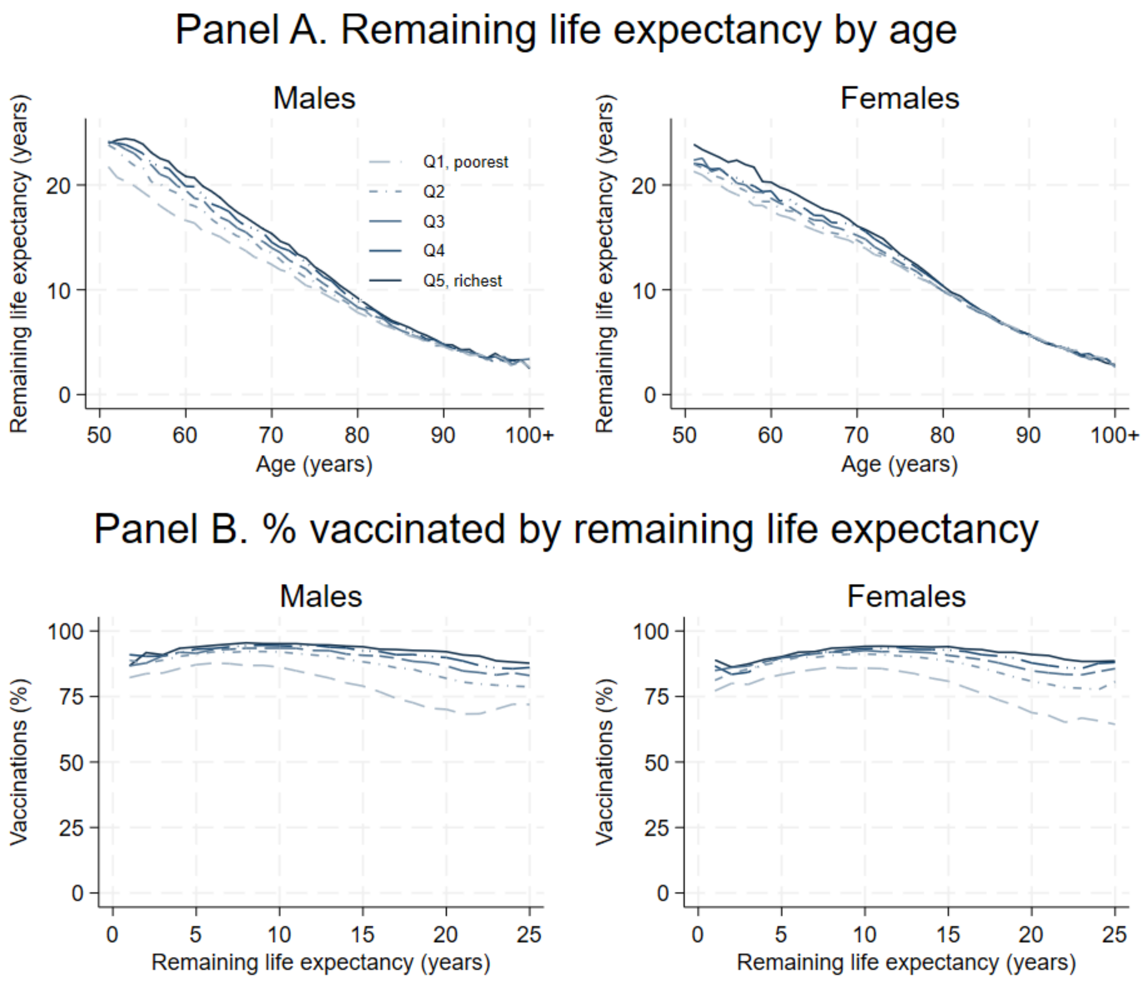

Supplementary Figure IV Panel A. Average remaining life expectancy by sex, age and income quintile. Panel B. Vaccination distribution across the poorest and richest income quintiles by remaining life expectancy.

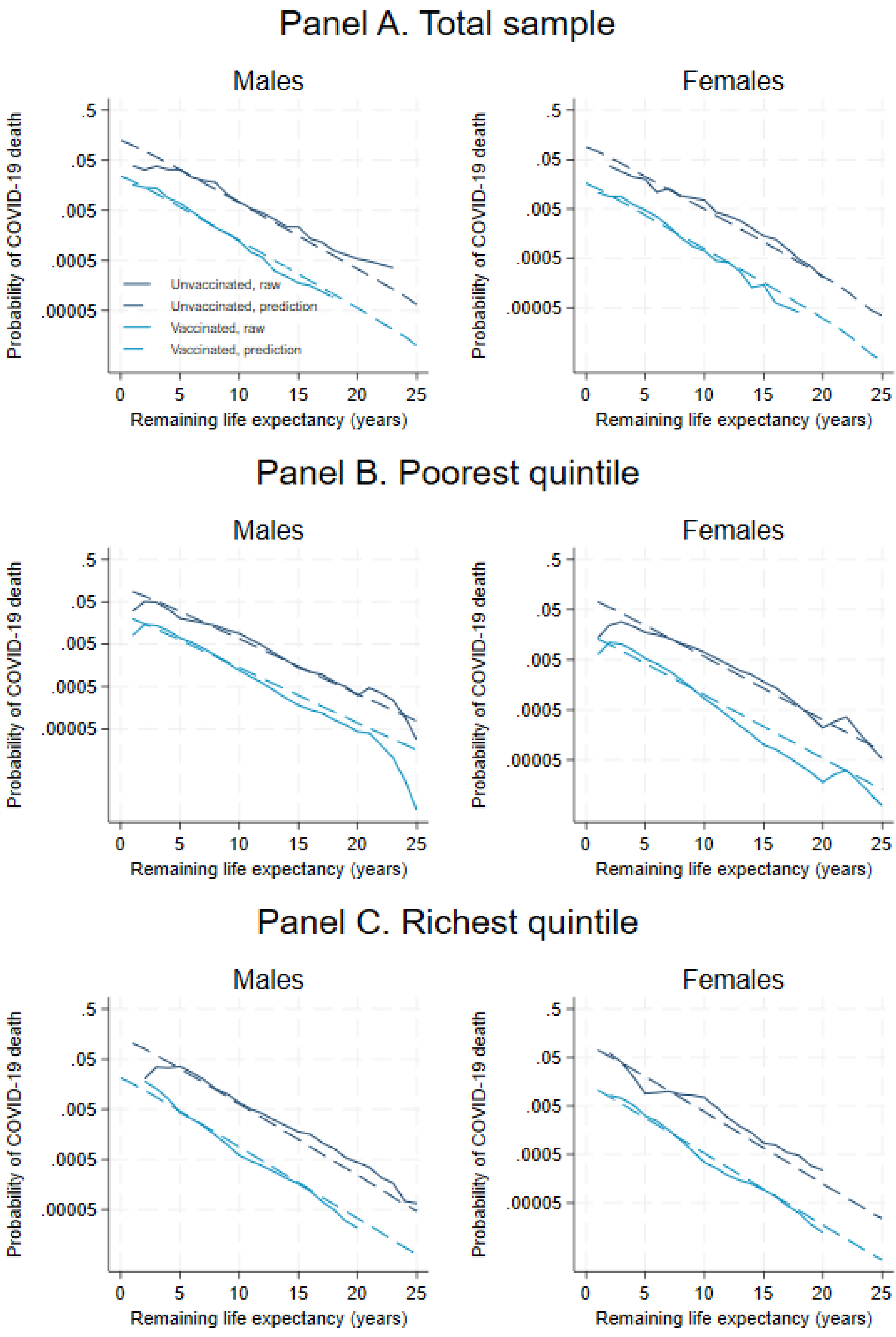

Supplementary Figure V Panel A. Average probability of COVID-19 death (presented on a logarithmic scale) for unvaccinated and vaccinated individuals predicted versus actual probability (raw) by remaining life expectancy for the entire sample by sex. Panel B. Average (smoothed) probability of COVID-19 death for unvaccinated and vaccinated individuals predicted versus actual probability (raw) by remaining life expectancy for the poorest quintile by sex. Panel C. Average (smoothed) probability of COVID-19 death for unvaccinated and vaccinated individuals predicted versus actual probability (raw) by remaining life expectancy for the richest quintile by sex. Note: Smoothing across life expectancy categories was necessary to comply with Statistics Netherlands anonymity retention guidelines.

More results and scenario analyses

Supplementary Table I Non-Linear decomposition of the income gap in COVID-19 mortality probability between the first four income quintiles and the richest quintile stratified by sex. Probabilities are rescaled by 10^3. Average probability of COVID-19 death is 1.1·10-3 for all males and 0.8·10-3 for all females.

	Vaccination (95% CI)	Remaining life expectancy (95% CI)	Unexplained (95% CI)	Total gap (95% CI)
Males				
Q1 (poorest)	0.14 (0.13-0.16)	0.70 (0.65-0.74)	0.20 (0.12-0.28)	1.04 (0.93-1.15)
Q2	0.06 (0.05-0.07)	0.38 (0.35-0.41)	0.21 (0.13-0.29)	0.65 (0.54-0.75)
Q3	0.03 (0.03-0.04)	0.23 (0.21-0.25)	0.18 (0.1-0.26)	0.45 (0.35-0.54)
Q4	0.01 (0.01-0.01)	0.09 (0.08-0.1)	0.16 (0.07-0.24)	0.26 (0.17-0.35)
Females				
Q1 (poorest)	0.12 (0.1-0.13)	0.29 (0.26-0.31)	0.22 (0.16-0.29)	0.63 (0.54-0.71)
Q2	0.03 (0.03-0.04)	0.16 (0.14-0.17)	0.17 (0.1-0.23)	0.36 (0.28-0.44)
Q3	0.01 (0.01-0.02)	0.1 (0.09-0.1)	0.14 (0.07-0.2)	0.25 (0.17-0.32)
Q4	0.003 (0.002-0.004)	0.03 (0.03-0.04)	0.05 (-0.02-0.11)	0.08 (0.01-0.15)

We conducted several scenario analyses to check the robustness of our findings in relation to the functional form of our remaining life expectancy measure (including a squared LE term), any remaining age gradient in income (restricting to ages 65+ only), and a placebo analysis (outcome is lung cancer instead of COVID-19). We also include two more checks – dropping the population we consider as "fully observed" in the main analysis but that had actually deregistered (lost to follow-up, LTFU; 4813 persons) and considering everyone who got vaccinated after 1-Aug as unvaccinated (2688 individuals). The latter case can be considered the lower bound of our results.

The placebo analysis serves as a robustness check if/how our main results are affected in case of any unobserved differences in health that are also correlated with vaccination uptake. Nevertheless, there are still a number of issues that make this placebo analysis less straightforward, which we illustrate with the example of lung cancer as an outcome.

First, there is a conceptual issue. Our analysis in which we use predicted life expectancy based on pre-COVID data is based on the fact that COVID-19 is a new cause of death, that did not exist during the period our prediction model is estimated on, and that COVID-19 mortality is known to be strongly correlated to pre-existing mortality risk. When repeating the analysis for other causes, we not only run into the fact that our health variable is partly based (or at least trained) on the outcome we are trying to predict, but also that a general life expectancy measure might have less predictive power for other specific causes than for COVID-19.

Second, for the analysis to be clearly interpretable as a robustness check, the alternative cause should show a similar mortality pattern by age and income as COVID-19, but at the same time be unrelated to COVID-19. As COVID-19 was a potential substitute cause for many other causes and/or an important contributing factor to other main causes of death (CBS RIVM, 2022), such unrelated causes are hard to find. Given the time trend in causes of death in the Netherlands, we picked lung cancer as one of the best candidates. Still, lung cancer patients who were infected with COVID-19 experienced more severe (COVID) outcomes (de Joode et al., 2020; Passaro et al., 2021).

Table II here shows a comparison between the coefficients of the regression models for COVID and lung cancer while Figure VI graphs a comparison between the decomposition results. From Table II we can see that the regression models for lung cancer perform less well in terms of explanatory power than the models for COVID-19. Looking at the odds ratios, we can see that the strength of the effects of remaining LE and vaccination is much smaller. The less strong effect of remaining LE indicates the issue that general LE might be a less good predictor for

some other causes than it is for COVID-19. While the coefficient for vaccination status is much smaller in the lung cancer models than in the COVID-19 models, it is significant. This could indicate that vaccination status is indeed correlated with unobserved health, but could also (partly) be explained by the relation between COVID-19 infection and severity of lung cancer outcomes. Figure VI shows that vaccination plays a smaller role in the decomposition of lung cancer deaths than COVID-19 deaths both in absolute and relative terms.

Supplementary Table II Regression results logistic regressions (presented in odds ratios) from various scenario analyses for the richest (Q5) and poorest (Q1) by sex. LTFU = lost to follow-up; removed 4813 individuals that moved out between 1-Aug and 31-Dec-2021. Unvaccinated after 1-Aug refers to a scenario where everyone who got vaccinated after 1-Aug was set to unvaccinated status (2688 individuals).

**Odds ratio (SE)**	**Males, Q1 (poorest)**					
Outcome	COVID-19	COVID-19	COVID-19	Lung cancer	COVID-19	COVID-19
Scenario	Base case	Functional form check	Ages 65+ only	Lung cancer	Dropped LTFU	Unvaccinated after 1Aug
Remaining life expectancy (LE)	.74*** (-0.005)	.769*** (-0.022)	.743*** (-0.007)	.849*** (-0.007)	.74*** (-0.005)	.738*** (-0.005)
LE squared		0.998 (-0.001)				
Vaccinated (0/1)	.208*** (-0.013)	.206*** (-0.013)	.211*** (-0.014)	.52*** (-0.044)	.207*** (-0.013)	.263*** (-0.017)
Observations	640466	640466	306571	640466	639579	640466
Pseudo R2	0.155	0.155	0.113	0.044	0.155	0.147
Akaike's Crit	13047.781	13047.725	11248.469	10629.303	13045.77	13180.118
Bayesian Crit	13081.891	13093.205	11280.369	10663.412	13079.876	13214.228
**Odds ratio (SE)**	**Males, Q5 (richest)**					
Remaining life expectancy (LE)	.72*** (-0.009)	.712*** (-0.028)	.711*** (-0.01)	.847*** (-0.009)	.72*** (-0.009)	.718*** (-0.008)
LE squared		1.001 (-0.002)				
Vaccinated (0/1)	.138*** (-0.016)	.139*** (-0.016)	.143*** (-0.017)	.444*** (-0.085)	.138*** (-0.016)	.17*** (-0.019)
Observations	640351	640351	306494	640351	639724	640351
Pseudo R2	0.219	0.219	0.17	0.06	0.219	0.215
Akaike's Crit	4948.232	4950.158	4591.795	4029.827	4947.594	4977.948
Bayesian Crit	4982.341	4995.638	4623.694	4063.936	4981.7	5012.058
**Odds ratio (SE)**	**Females, Q1 (poorest)**					
Remaining life expectancy (LE)	.747*** (-0.006)	.813*** (-0.029)	.753*** (-0.008)	.86*** (-0.007)	.747*** (-0.006)	.744*** (-0.006)
LE squared		.996** (-0.002)				
Vaccinated (0/1)	.172*** (-0.013)	.168*** (-0.013)	.18*** (-0.015)	.506*** (-0.047)	.172*** (-0.013)	.219*** (-0.017)
Observations	683646	683646	347624	683646	682983	683646
Pseudo R2	0.16	0.161	0.128	0.036	0.16	0.15
Akaike's Crit	9065.256	9060.852	7776.931	8542.671	9064.212	9178.457
Bayesian Crit	9099.561	9106.593	7809.208	8576.977	9098.514	9212.762
**Odds ratio (SE)**	**Females, Q5 (richest)**					
Remaining life expectancy (LE)	.712*** (-0.011)	.748*** (-0.041)	.705*** (-0.013)	.854*** (-0.01)	.712*** (-0.011)	.711*** (-0.01)
LE squared		0.997 (-0.003)				
Vaccinated (0/1)	.14*** (-0.018)	.138*** (-0.018)	.146*** (-0.02)	.515*** (-0.101)	.14*** (-0.018)	.173*** (-0.022)
Observations	683501	683501	347513	683501	683057	683501
Pseudo R2	0.224	0.224	0.182	0.048	0.224	0.218
Akaike's Crit	3502.965	3504.038	3256.436	3701.838	3502.693	3528.654
Bayesian Crit	3537.27	3549.778	3288.712	3736.143	3536.996	3562.959

*** p<.01, ** p<.05, * p<.1

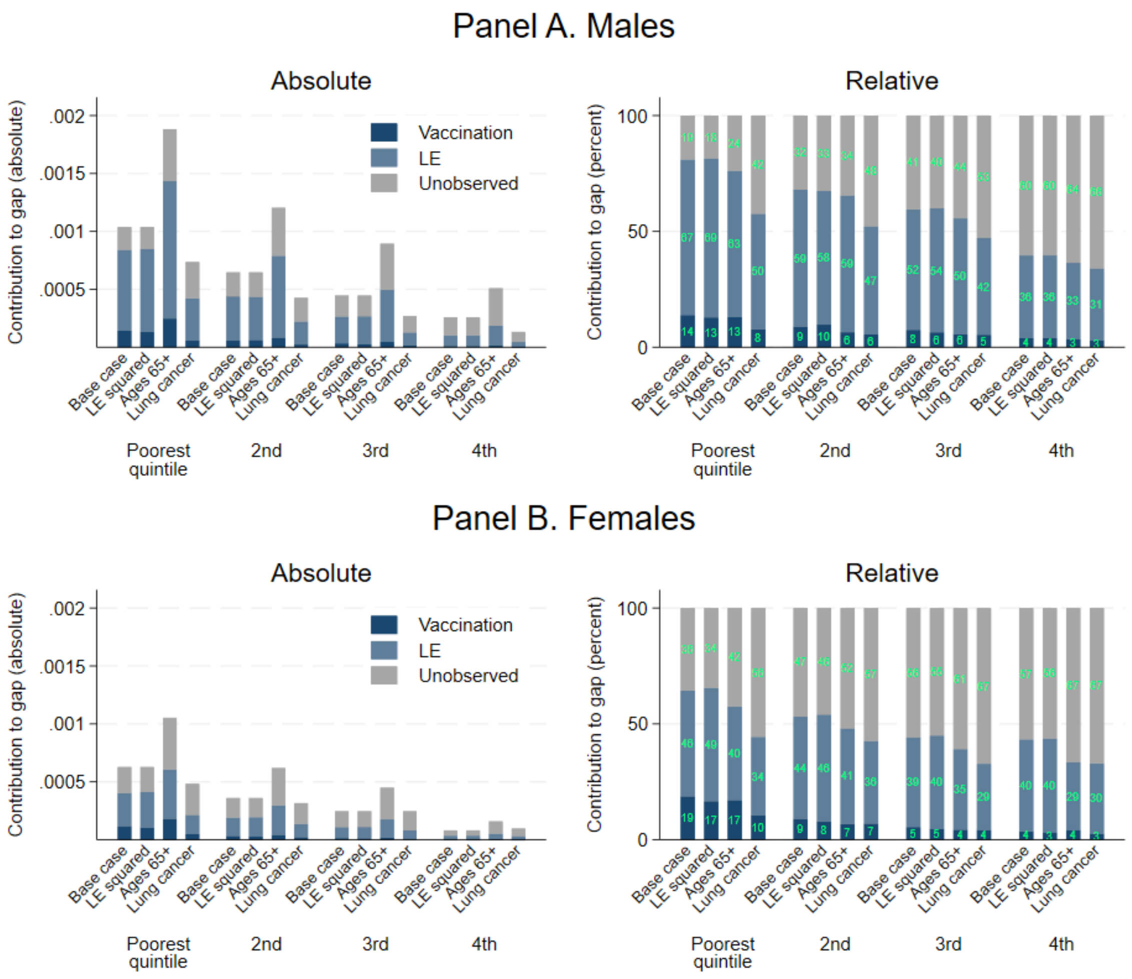

Supplementary Figure VI Decomposition results from various scenario analyses compared to base case – LE squared, ages 65+, lung cancer.

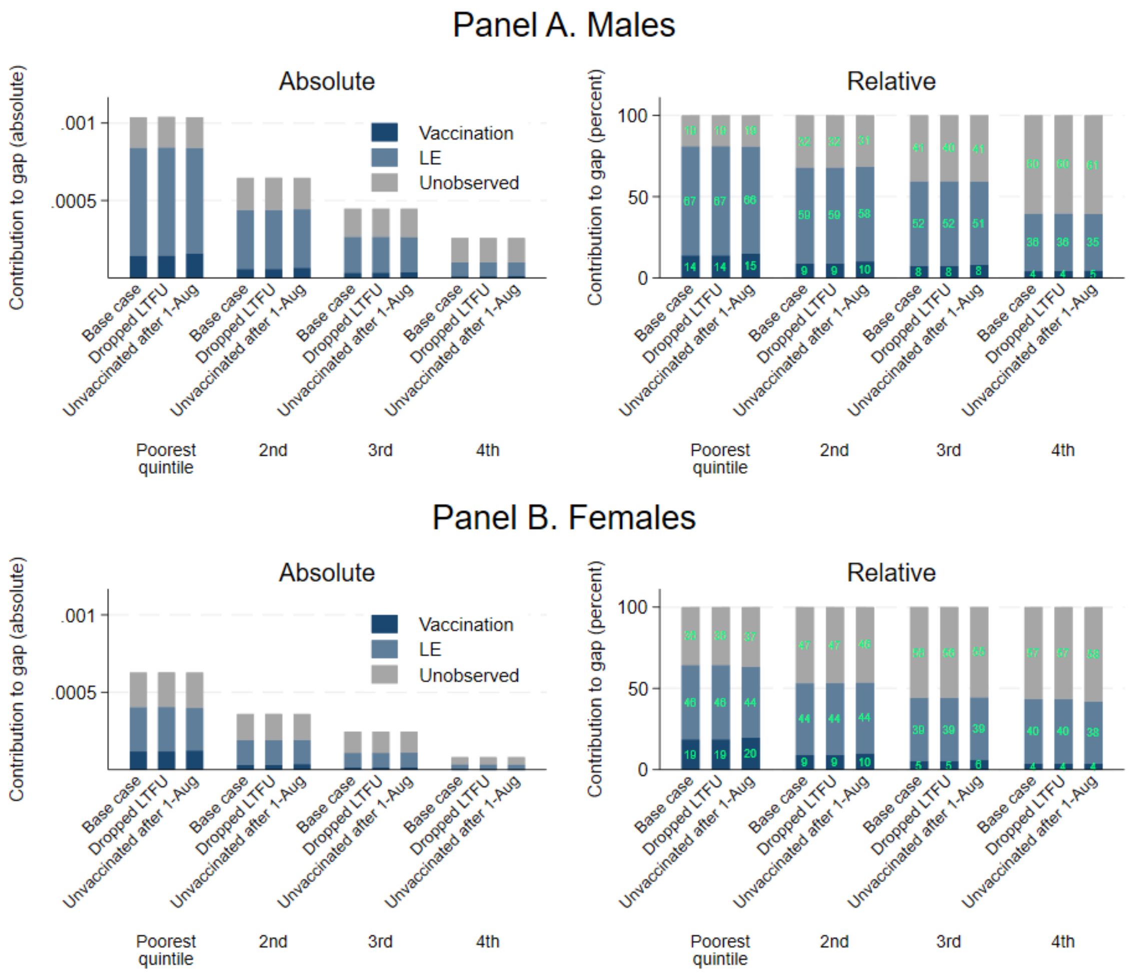

Supplementary Figure VII Decomposition results from various scenario analyses compared to base case – no LTFU and a lower bound analysis.

Non-linear decomposition

The best-fitting and most parsimonious model is selected to perform a decomposition of the income inequality gap. Decompositions are used to differentiate the contribution of endowment (differences in distribution of predictors) and coefficients (differences in magnitude of coefficients/response) to inequalities between two groups. A popular decomposition is the Oaxaca-Blinder decomposition (Blinder, 1973; Oaxaca, 1973), which is a linear decomposition. However, considering the binary nature of the outcome variable, death from COVID-19, we opted for a non-linear decomposition (Powers et al., 2011). The general formula for decomposing differences between the poorest and richest quintiles is as follows:

$$\bar{Y}_{Q1} - \bar{Y}_{Q5} = \overline{F(X_{Q1}\beta_{Q1})} - \overline{F(X_{Q5}\beta_{Q5})} \quad (1)$$

$$= \left\{\overline{F(X_{Q1}\beta_{Q1})} - \overline{F(X_{Q5}\beta_{Q1})}\right\} + \left\{\overline{F(X_{Q5}\beta_{Q1})} - \overline{F(X_{Q5}\beta_{Q5})}\right\} \quad (2)$$

Here, we show the decomposition formula for the inequality gap between the poorest quantile (Q1) and the richest quintile (Q5). The mean difference in the outcome Y (death from COVID-19) can be expressed as the difference in the functions defining Y_{Q1} and Y_{Q5} (see equation (1)). With manipulation, we get equation (2), where the first pair of brackets contains the endowment, and the second pair of brackets contains the coefficient effect. In other words, the endowment effect pertains to the differences in outcome related to a change in the distribution of X as one goes from the distribution in the richest quintile to the poorest. In this instance for example, X is a vector consisting of remaining life expectancy and vaccination status and β is a vector of coefficients. The coefficient effect reflects the differences in outcome related to varying coefficients across income groups. This decomposition is performed separately for males and females, and for differences between the richest quintile and the four poorer quintiles.

Remaining life expectancy (LE)

As is shown in Supplementary Figure II, we started off by gathering all individuals who were at least 50 years of old as of 1-Jan-2020, who also had registered income in the previous year. The reason for this was that the model that predicts the remaining life expectancy metric works with 1-year lagged covariates on healthcare use and income, and we wanted to avoid using covariates biased by the pandemic. The model can only predict LE for ages 50+. As such, the initial LE value that was calculated by the model was individual-level LE as of 1-Jan-2020. Since it was easier to recalculate LE on an annual basis and because we were interested in 2021 values, we used the following method to re-calculate remaining LE as of 1-Jan-2021 for each individual (i):

$$LE_i(2021) = \frac{\int_{t=2021}^{2070} S_i(t)}{S_i(2021)}$$

Where S_i is the survival curve for each individual at distinct times.

Calibration

The calibration test the coefficients γ_0 and γ_1, where the dependent variable is $\widetilde{F}_i(t)$, are the pseudo-observations (observed cumulative failure probabilities corrected for censoring) (Royston, 2014):

$$\widetilde{F}_i(t) = \gamma_0 + \gamma_1 * \ln\widehat{H}(t, x_i) = \gamma_0 + \gamma_1 * (\ln\widehat{H}_0(t) + x_i\beta)$$

- Calibration intercept – the closer to 0, the better. A negative/positive value means we have a systematic over/under prediction, stemming from overall difference in the true baseline survival between training and prediction datasets. A positive intercept would suggest that the mortality rate at the selected time is exp(γ_0) higher in the validation dataset.
- Calibration slope – the closer to 1 means we have good agreement between estimated and observed individual event probabilities estimated at different time points.
- Harrell C-Index (Newson, 2010) which can take on values from zero to one: the higher, the better. If the model is any good then observations with a shorter time-to-event T, should also have a higher risk score. The index itself measures how often in a two-paired observations setting does this hold true plus half the probability that the hazards in the randomly two paired observations are equal.

Supplementary Table III Survival model performance measures on the validation dataset (i.e., people alive at start of 2019 and followed up until 31-Dec-2019).

					P-value tests with constraints	
Income decile	**male**	**Calibration intercept (t=end of 2019)**	**Calibration slope (Royston, 2014)**	**C-Index (Newson, 2010)**	**Test1 (intercept=0 when slope==1)**	**Test2 (slope=1, when intercept==0)**
1	0	0.177	0.945	0.837	0	0.0008
2	0	0.246	0.944	0.851	0	0.0007
3	0	0.194	0.981	0.869	0	0.2402
4	0	0.206	0.952	0.878	0	0.0028
5	0	0.129	0.930	0.881	0	0
6	0	0.194	0.932	0.889	0	0.0001
7	0	0.138	0.945	0.894	0	0.0017
8	0	0.197	0.947	0.894	0	0.0051
9	0	0.141	0.969	0.896	0	0.101
10	0	0.203	0.963	0.906	0	0.041
1	1	0.083	1.028	0.808	0	0.056
2	1	0.052	1.009	0.834	0	0.500
3	1	0.063	1.012	0.858	0	0.416
4	1	0.047	0.984	0.868	0	0.291
5	1	0.093	1.000	0.875	0.0003	0.985
6	1	0.033	0.978	0.876	0	0.166
7	1	0.079	0.992	0.880	0	0.639
8	1	0.038	0.982	0.888	0.098	0.283
9	1	0.090	1.005	0.890	0	0.775
10	1	0.075	0.995	0.894	0.0004	0.794

The models generally seem to be well calibrated, although not perfectly, but that seems to be the case irrespective of income decile. The slopes are all close to 1. For men, the models exhibit somewhat better calibration as the intercepts are closer to 0 than for women. Given that the decomposition is stratified by sex this should not influence our decomposition. We seem to have overall a good discrimination (C-index) across all income groups (>80%), but there is indeed some evidence that at the richest deciles the models are a bit better at producing a high risk score when the event (death) is sooner. However, this could at least in part be due to more ties in the risk score prediction in the poorer groups, seeing how our survival models predominantly categorical values (Hartman et al., 2023). In any case, there is no evidence of an income gradient in the (mis)calibration.

References

Blinder, A. S. (1973). Wage Discrimination: Reduced Form and Structural Estimates. *The Journal of Human Resources*, *8*(4), 436. https://doi.org/10.2307/144855

CBS RIVM. (2022). *Sterfte en oversterfte in 2020 en 2021 | CBS*. https://www.cbs.nl/nl-nl/longread/rapportages/2022/sterfte-en-oversterfte-in-2020-en-2021

de Joode, K., Dumoulin, D. W., Tol, J., Westgeest, H. M., Beerepoot, L. V., van den Berkmortel, F. W. P. J., Mutsaers, P. G. N. J., van Diemen, N. G. J., Visser, O. J., Oomen-de Hoop, E., Bloemendal, H. J., van Laarhoven, H. W. M., Hendriks, L. E. L., Haanen, J. B. A. G., de Vries, E. G. E., Dingemans, A. M. C., van der Veldt, A. A. M., van Loenhout, C. J., van der Leest, C. H., … Bootsma, G. P. (2020). Dutch Oncology COVID-19 consortium: Outcome of COVID-19 in patients with cancer in a nationwide cohort study. *European Journal of Cancer*, *141*, 171–184. https://doi.org/10.1016/j.ejca.2020.09.027

Hartman, N., Kim, S., He, K., & Kalbfleisch, J. D. (2023). Pitfalls of the concordance index for survival outcomes. *Statistics in Medicine*, *42*(13), 2179–2190. https://doi.org/10.1002/SIM.9717;PAGE:STRING:ARTICLE/CHAPTER

Newson, R. B. (2010). Comparing the predictive powers of survival models using Harrell's C or Somers' D. *Stata Journal*, *10*(3), 339–358. https://doi.org/10.1177/1536867X1001000303;PAGE:STRING:ARTICLE/CHAPTER

Oaxaca, R. (1973). Male-Female Wage Differentials in Urban Labor Markets. *International Economic Review*, *14*(3), 693. https://doi.org/10.2307/2525981

Passaro, A., Bestvina, C., Velez Velez, M., Garassino, M. C., Garon, E., & Peters, S. (2021). Severity of COVID-19 in patients with lung cancer: evidence and challenges. *Journal for ImmunoTherapy of Cancer*, *9*(3), 2266. https://doi.org/10.1136/JITC-2020-002266

Powers, D. A., Yoshioka, H., & Yun, M.-S. (2011). mvdcmp: Multivariate decomposition for nonlinear response models. In *The Stata Journal* (Vol. 11, Number 4).

Royston, P. (2014). Tools for checking calibration of a Cox model in external validation: Approach based on individual event probabilities. *Stata Journal*, *14*(4), 738–755. https://doi.org/10.1177/1536867X1401400403;WEBSITE:WEBSITE:SAGE;ISSUE:ISSUE:DOI
